# Risk management and empirical study of the doctor-patient relationship: based on 1790 litigation cases of medical damage liability disputes in China

**DOI:** 10.1186/s12913-024-10952-x

**Published:** 2024-04-25

**Authors:** Hui Li, Limin Li, Tong Liu, Meiqiong Tan, Wanwan He, Yuzhu Luo, Xuerong Zhong, Liping Zhang, Jiangjie Sun

**Affiliations:** 1 School of Health Care Management, Anhui Medical University, 230032 Hefei, China; 2 The Second Clinical Medical College, Anhui Medical University, 230032 Hefei, China; 3 School of Marxism, Anhui Medical University, 230032 Hefei, China; 4 School of Management, Hefei University of Technology, 230039 Hefei, China

**Keywords:** Doctor-patient relationship, Medical damage, Liability disputes, Risk management, Empirical analysis, Compensation

## Abstract

**Background:**

Compensation for medical damage liability disputes (CMDLD) seriously hinders the healthy development of hospitals and undermines the harmony of the doctor-patient relationships (DPR). Risk management in the DPR has become an urgent issue of the day. The study aims to provide a comprehensive description of CMDLD in China and explore its influencing factors, and make corresponding recommendations for the management of risks in the DPR.

**Methods:**

This study extracted data from the China Judgment Online - the official judicial search website with the most comprehensive coverage. Statistical analysis of 1,790 litigation cases of medical damage liability disputes (COMDLD) available from 2015 to 2021.

**Results:**

COMDLD generally tended to increase with the year and was unevenly distributed by regions; the compensation rate was 52.46%, the median compensation was 134,900 yuan and the maximum was 2,234,666 yuan; the results of the single factor analysis showed that there were statistically significant differences between the compensation for different years, regions, treatment attributes, and trial procedures (*P* < 0.05); the correlation analysis showed that types of hospitals were significantly negatively associated with regions (*R*=-0.082, *P* < 0.05); trial procedures were significantly negatively correlated with years (*R*=-0.484, *P* < 0.001); compensat- ion was significantly positively correlated with years, regions, and treatment attributes (*R* = 0.098–0.294, *P* < 0.001) and negatively correlated with trial procedures (*R*=-0.090, *P* < 0.01); regression analysis showed that years, treatment attributes, and regions were the main factors affecting the CMDLD (*P* < 0.05).

**Conclusions:**

Years, regions, treatment attributes, and trial procedures affect the outcome of CMDLD. This paper further puts forward relevant suggestions and countermeasures for the governance of doctor-patient risks based on the empirical results. Including rational allocation of medical resources to narrow the differences between regions; promoting the expansion and sinking of high-quality resources to improve the level of medical services in hospitals at all levels; and developing a third-party negotiation mechanism for medical disputes to reduce the cost of medical litigation.

## Introduction

Against the background of COVID-19, China once again saw a harmonious situation in which “health is related to life”, and the DPR was on the rise [1]. But the short-lived peace was then shattered when Hu Shuyun, a cardiologist in Jishui County, Jiangxi Province, was attacked and injured by an assailant during a ward check on January 26, 2021, and later died after an ineffective rescue attempt. In recent years, there have been incidents of medical violence and injuries to doctors [2, 3]. At a time when the entire population is united in the fight against the epidemic, and when the “messengers in white” are traveling thousands of miles to save the lives of the public, the occasional conflict between doctors and patients seriously interferes with the responsibility and social commitment of the medical and nursing community, seriously hinders the construction of a healthy medical environment, and the risks to doctors and patients need to be addressed.

A harmonious DPR is the basis for a successful therapeutic outcome [4]. However, the ‘disease-centric’ model of health that has been dominated by biomedical thinking since the mid-20th century is deeply entrenched and the needs of patients have been severely neglected. As a result, the symptoms of the disease are temporarily relieved while the root causes of the problems in the DPR remain [5]. In the UK, a study by a health service journal and UNISON found that 181 NHS trusts in England reported a whopping 56,435 incidents of physical assault on staff in 2016–2017 [6]. A survey of 16,327 practicing physicians in Australia found that 71% reported having experienced verbal or written assault and 32% had experienced physical assault [7]. The DPR in China is equally unpromising, with the White Paper on Doctor Practice in China showing that 62% of doctors and patients have varying degrees of medical disputes [8]. The literature shows that the current DPR is generally perceived as poor by both doctors and patients [9–11].

There is a large body of literature on how to improve patient satisfaction and thus ease the patient-practitioner relationship. Wang M et al. argued, at the level of the healthcare provider, that by improving hospital management, hospital litigation costs can be reduced, potential harm to patients reduced, and patient and staff satisfaction increased, thus improving the DPR [12]. In the context of COVID-19, Xu B noted that improving doctor-patient communication, medical technology, and patients’ medical knowledge may help improve the DPR [4]. In addition, as eHealth plays an increasingly important role in public health services, Wynn R noted the need to consider how eHealth can leverage and integrate aspects of the traditional DPR to improve services and engage patients [13].

Another part of the literature investigates the mechanisms for resolving medical disputes. Medical disputes are usually disputes between doctors and patients who do not agree on the consequences and causes of medical treatment, when they may seek accountability or compensation for damages from the health administration or the judiciary [14]. Litigation is the legal way to resolve medical disputes, but it has the disadvantages of consuming the time and energy of both doctors and patients, complicated trial procedures, and high costs. Research in the literature has found that many countries and regions tend to adopt an Alternative Dispute Resolution model for resolving medical disputes. Ferris LE et al. study points out that it is believed that doctor-patient disputes should be considered first and foremost to be resolved in an alternative form to save judicial resources effectively [15]. Yee F compared mediation, arbitration, and litigation in medical disputes and concluded that mediation has the advantage of being the most efficient and least damaging to the interests of both doctors and patients, and therefore should be widely promoted [16]. Moore J et al. proposed the Communication-and-Resolution Programs model after practice, which focuses on communication with patients and their families after an adverse event can bring them a better psychological feeling and facilitate the resolution of the adverse event [17].

At the same time, the improvement of medical quality has become the focus of attention from all walks of life and is of great significance in easing the DPR and preventing disputes between them. Valls Martinez MdC et al. used structural equation modeling to explore the relationship between service quality and satisfaction from the patient’s perspective, using an Iranian hospital as a study, and showed that optimizing service quality contributed to patient satisfaction and that patients cared most about hospital hygiene and humanistic care [18]. A study by Thawesaengskulthai N et al. found that the development of service quality measurement models should take into account not only specific situations such as location, but also the nationality and demographics of patients, and that patients’ perceived quality of healthcare services changed over time [19]. Furthermore, a study by Hanefeld J et al. further suggests that the complexity of quality of care requires not only improving technical quality but also patient-provider acceptability, responsiveness, and trust levels, which in turn improves understanding of all attributes of health system quality and their interrelationships and helps to expand access to essential health interventions [20].

Contemporary research has yielded good results in all aspects of the current state of DPR, risk management of DPR, mechanisms for resolving doctor-patient disputes, and improving healthcare quality. Since 2021, the risk of DPR has been gradually judicialized, but no research topic on judicial data on the risk of the DPR has been found, so this study uses authoritative datasets extracted from national-level adjudication documents to provide a comprehensive description of CMDLD in China and to explore its influencing factors, with a view intending to providing references for easing DPR, improving healthcare quality and improving the healthcare environment.

## Data and methods

### Study design and samples

We conducted a comprehensive search of data from 2015 to 2021 using the China Judgment Online, a public database of court cases concluded. In the database, the full text is used as the search field, “medical damage liability dispute” is used as the search term, civil is used as the cause of the case, the civil case is used as the case type, judgment is used as the type of instrument, and years of medical damage liability dispute jurisprudence are 2015–2021. The inclusion criteria are as follows: (a) cases in which the subject matter is “dispute over liability for medical damage”; (b) cases in which the plaintiff is the patient; (c) cases in which the defendant is a medical institution. Exclusion criteria: (a) cases with a cause of action other than dispute over liability for medical damage such as dispute over victimization of a labor provider, traffic accident dispute, and accidental injury insurance contract, etc.; (b) cases whose time of closure and adjudication is not between 2015 and 2021; (c) cases in which the plaintiff is a medical institution or the defendant is a patient; and (d) cases in which the adjudication, judgment, etc. is dismissed, conciliated, or withdrawn. (e) cases in which key information such as the name of the hospital, the time of the dispute, and the amount of compensation is missing.

The following data were extracted from the included studies. Years, regions (the Eastern region, the Middle region, and the Western region), types of hospitals (township health center, county hospitals, and city/provincial hospitals), patients’ purposes for appeal (truth over claims, claims over the truth), treatment attributes (low cost-effectiveness of treatment, poor treatment effect, complications of treatment, treatment leading to disability, and treatment leading to death), procedures (the first trial, the second trial, and the retrial), the outcome of the financial compensation (yes or no), and compensat- ion amount. Four researchers were assigned to each case to extract key information, double-blindly enter the extracted information into the software, and check for errors to ensure the accuracy of the data.

### Methods

Data from the sample distribution were sorted using Excel software, processed using SPSS 26.0 [21–23], and the distribution of compensation amounts by year was described in R language [24, 25] in the form of median M (interquartile spacing). The Mann-Whitney U test and the Kruskal-Wallis H test were used to compare the dichotomous and multicategorical variables between groups, respectively, at the test level (α = 0.1). Correlations between the dependent and some of the independent variables were analyzed by Spearman, and finally, variables that were statistically significant in the one-way analysis (*p* < 0.1) were screened out and linear regression analysis was applied to explore the factors influencing the award of medical damage liability disputes (α = 0.05).

## Results

### Overall situation of COMDLD

**Fig. 1:**
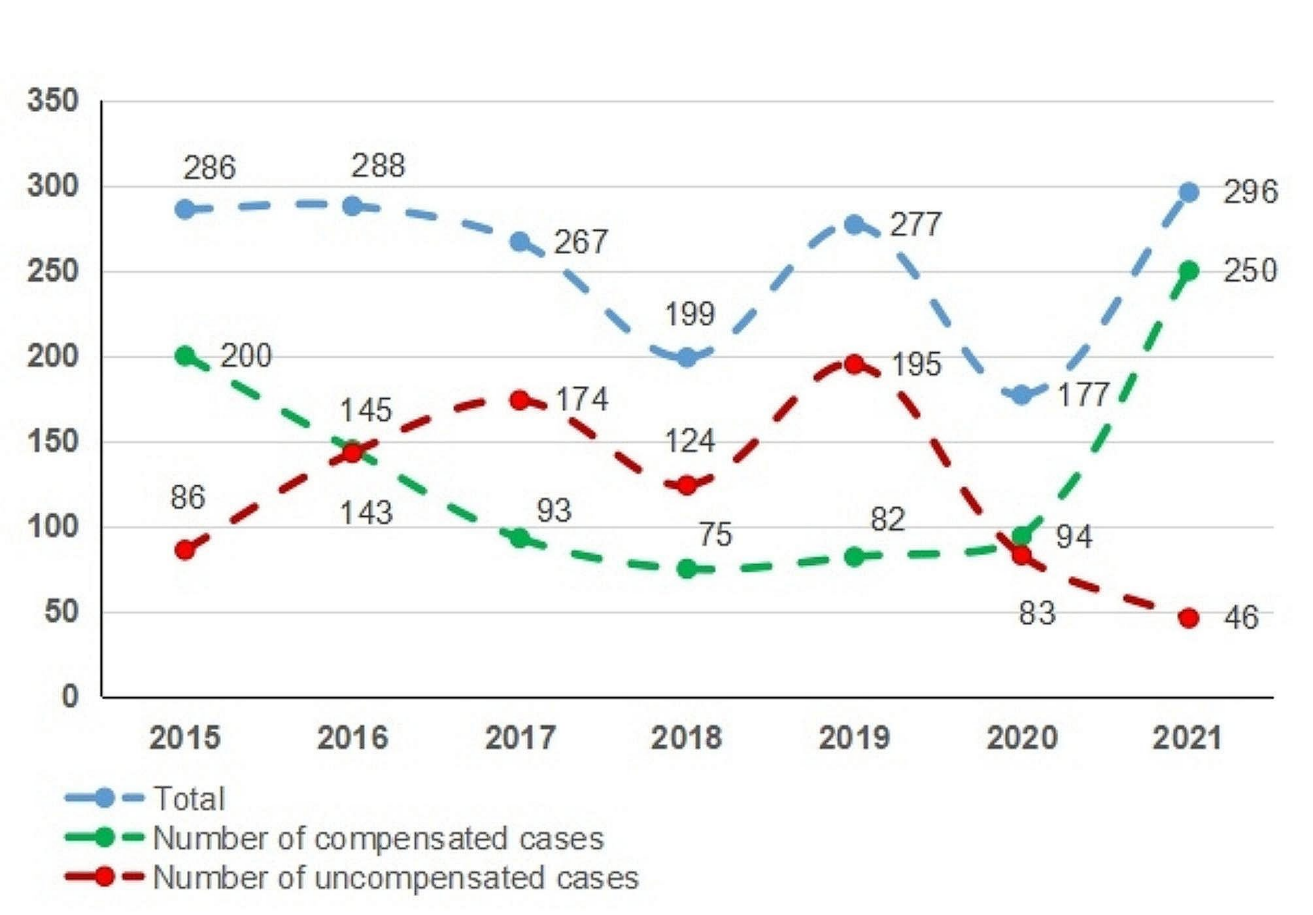
Year distribution of COMDLD (2015–2021)

COMDLD included in the criteria for 2015–2021 is 1,790. The number of cases generally trended upwards overall, with a maximum of 296 cases in 2021; the number of awarded cases trended steadily upwards; and the number of unawarded cases trended upwards until 2019 and downwards after 2019. (See Fig. 1)

**Fig. 2:**
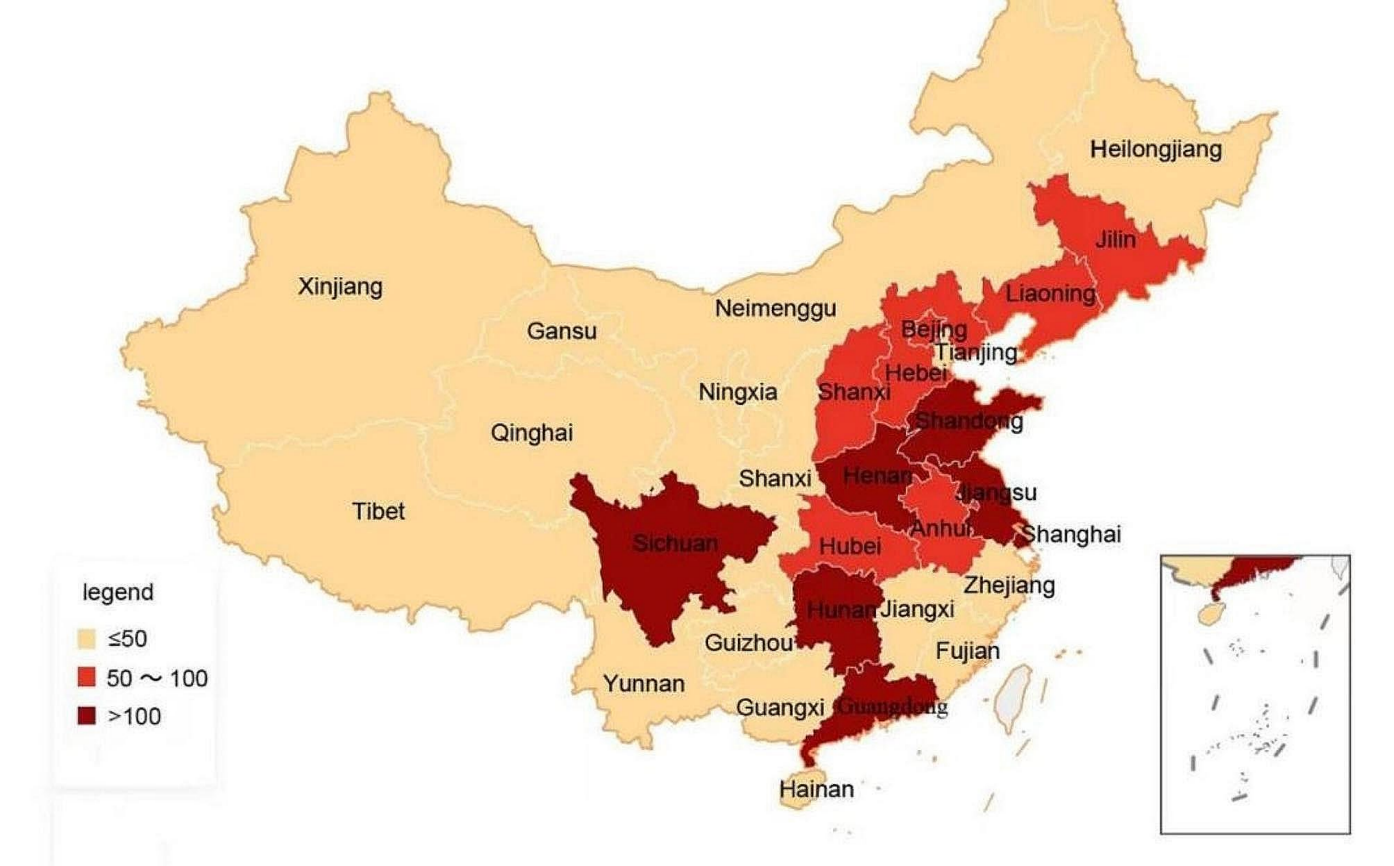
Regional distribution of COMDLD

Of the 1,790 cases studied, a total of 22 provinces, five autonomous regions, and four municipalities directly under the central government were involved. The number of COMDLD was unevenly distributed across provinces, autonomous regions, and municipalities directly under the central government. Based on the combination of the level of economic development and geographical location, there are three main regions: the Eastern region, the Middle region, and the Western region. The Eastern region is the most economically developed, followed by the Middle region, and the Western region is less developed. The top five provinces (cities) where medical damage liability disputes occurred were Henan Province, Jiangsu Province, Shanghai, Hunan Province, and Guangdong Province, accounting for 160 (8.94%), 121 (6.76%), 111 (6.20%), 110 (6.15%) and 104 (5.81%) respectively. The provinces (cities) in this category are located in the Eastern and Middle regions, have relatively abundant medical resources, are densely populated, have a high number of attendances, and therefore have a high number of COMDLD. (See Fig. 2)

### Distribution of CMDLD

**Fig. 3:**
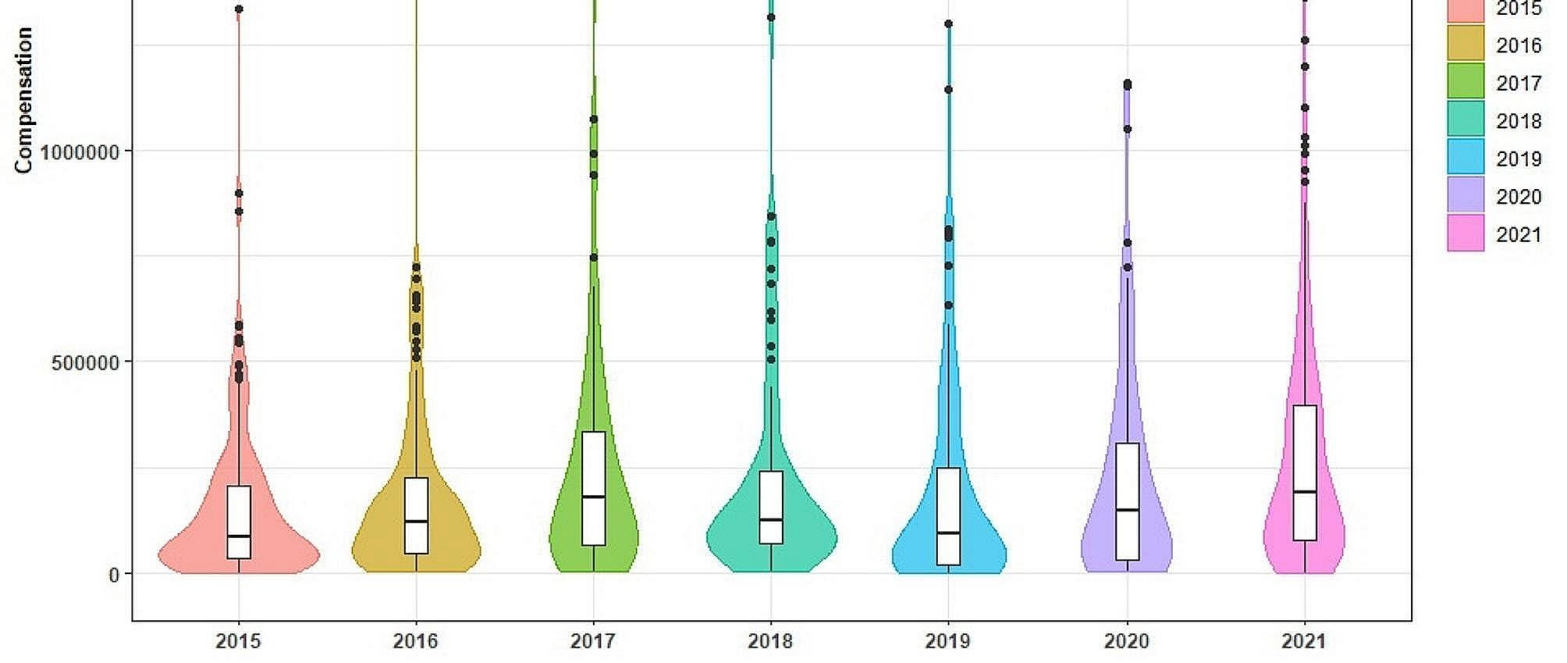
Distribution of CMDLD(2015–2021)

We used the ggplot package in R studio 2022.12.0 to plot violin plots overlaid with box line plots. Violin plots are used to show the shape of the distribution of multiple sets of data. Of the 1,790 COMDLD heard by the courts, the number of medical damage liability dispute cases in which compensation was awarded totaled 939, with a case award rate of 52.46% and a median award of 134,900 yuan with a maximum value of 2,234,666 yuan. Although CMDLD fluctuated overall, the median award overall continued to trend upwards across all years. (See Fig. 3)

### Factors that influence CMDLD

By testing the normality of the 939 cases of CMDLD, it was found that the compensation did not follow a normal distribution, so the Mann-Whitney U test and Kruskal - Wallis H test were used to compare the compensation in different years, regions, types of hospitals, patients’ purposes for appeal, treatment attributes, and trial procedures.

Among the different year distributions, 2021 had the highest number of CMDLD, 250 cases, with a median of 191,897.83 yuan. The Kruskal-Wallis H-test found that the difference in CMDLD between the different year distributions was statistically significant (H = 38.251, *p* < 0.001); among the different regional distributions, the Eastern region had more cases that generated CMDLD, with 418 cases and a median of 111,463.00 yuan. The Kruskal-Wallis H-test revealed a statistically significant difference in CMDLD between the different regional distributions (H = 9.692, *p* < 0.05); Among the different hospital types, city/provincial hospitals generated more cases of CMDLD, with 698 cases, followed by county hospitals and the least by township health centers. The Kruskal-Wallis H-test found that the difference between the different types of hospitals was not statistically significant (H = 5.150, *p* < 0.1); according to the purpose of the patient’s appeal, The Mann-Whitney U test showed no statistically significant difference in CMDLD by patient’s purpose of appeal (Z=-1.355b, *p* > 0.1); According to the different treatment attributes, the number of cases with medical damage liability disputes arising from treatment leading to death and the amount of compensation was the highest, with a total of 399 cases and a median of 186,300.00 yuan. According to the Kruskal-Wallis H-test, the difference in CMDLD between different treatment attributes was statistically significant (H = 90.491, *p* < 0.001); divided according to different trial procedures, the second trial had the highest number of cases generating compensation for medical damage liability disputes, with a total of 701 cases, while the first trial had the highest number of cases generating compensation for medical damage liability disputes The highest amount was awarded in the first trial, with a median of 183,497.90 yuan. The Kruskal-Wallis H-test showed that the difference in CMDLD between the different trial procedures was statistically significant (H = 9.660, *p* < 0.01). (See Table 1)

**Table 1:** Analysis of influencing factors of CMDLD

Items		Total cases	Number of compensated cases (%)	Amount of compensation	
				Amount (yuan)[M(P_25_, P_75_)	Statistics
Years	2015	286	200(69.93%)	85,700.00 (33,750.00,213,825.00)	H = 38.251^a^ (*P* < 0.001)
	2016	288	145(50.35%)	120,400.00(43,900.00,227,047.11)	
	2017	267	93(34.83%)	177,611.00 (61,286.00,336,450.00)	
	2018	199	75(37.69%)	125,832.00 (68,634.00,242,106.00)	
	2019	277	82(29.60%)	93,329.50 (17,562.75,264,800.00)	
	2020	177	94(53.11%)	148,987.55 (30,810.08,313,946.55)	
	2021	296	250(84.46%)	191,897.83 (76,179.39,400,235.91)	
Regions	Eastern	864	418(48.38%)	111,463.00(30,000.00,304428.90)	H = 9.692^a^ (*P* = 0.008)
	Middle	632	353(55.85%)	130,069.00(56,839.50,274042.00)	
	West	294	168(57.14%)	151,718.00(86,853.25,346,510.54)	
Types of Hospitals	Township health center	95	53(55.79%)	90,100.00 (31,840.05,192,366.85)	H = 5.150^a^ (*P* = 0.076)
	County Hospital	328	188(57.32%)	140,215.75(57,697.03,279,120.48)	
	City/provincial hospital	1,367	698(51.06%)	135,258.00(46,790.75,317,765.41)	
Patients’ purposes for appeal	Truth over claims	274	114(41.61%)	107,810.22(27,412.50,301,975.00)	Z=-1.355^b^ (*P* = 0.175)
	Claims over the truth	1,516	825(54.42%)	138,500.00(49,566.31,302,877.17)	
Treatment Properties	Low Cost-effectiveness of treatment	68	19(27.94%)	20,000.00(6,100.00,296,051.35)	H = 90.491^a^ (*P* < 0.001)
	Poor treatment effect	165	64(38.79%)	30,000.00(10,000.00,92,502.07)	
	Complications of treatment	161	79(49.07%)	54,000.00(26,121.95,158,687.50)	
	Treatment leading to disability	636	378(59.43%)	110,663.50(47,280.25, 294,271.03)	
	Treatment leading to death	760	399(52.50%)	186,300.00(86,880.71,370,033.00)	
Procedures	First trial	260	222(85.38%)	183,497.90(60,159.73,357,006.40)	H = 9.660^a^ (*P* = 0.008)
	Second trial	1,473	701(47.59%)	120,400.00(39,850.00,262,293.50)	
	Retrial	57	16(28.07%	127,525.50(70,000.00,555,213.00)	

Note: ^a^ refers to the Mann-Whitney U rank sum test for groups of two independent samples; ^b^ refers to the Kruskal-Wallis H rank sum test for groups of multiple samples

### Correlation analysis

Before the regression analysis, a correlation analysis was conducted between the dependent variable and some of the independent variables, and the Spearman correlation matrix was calculated using SPSS 26.0 software and it was concluded; there was a high correlation between the indicators. It can be seen that the correlation between the partial independent variables and the dependent variable, to a certain extent, verifies the reasonableness of the selection of the independent variables in this paper. Moreover, none of the correlation coefficients between the independent variables exceeded the critical value that might lead to the problem of multicollinearity, and could better meet the requirements of regression analysis. (See Table 2)

**Table 2:** Correlation analysis of compensation cases in medical damage liability disputes

Variables	Years	Regions	Types of Hospitals	Treatment Properties	Procedures	Compensation
Years	1					
Regions	0.004	1				
Types of Hospitals	0.019	-0.082*	1			
Treatment Properties	-0.047	0.033	-0.017	1		
Procedures	-0.484**	0.009	0.025	0.042	1	
Compensation	0.169**	0.098**	0.042	0.294**	-0.090**	1

Note: ** and *indicate the significant *p* value at the 1% and 5%

### Linear regression analysis

Compensation based on COMDLD, the influencing factors that were statistically significant (*p* < 0.1) were selected as independent variables to enter the multi-factor analysis. The independent variables were years, regions, types of hospitals, treatment attributes, and trial procedures. Due to the large value of compensation, the logarithm was taken to participate in the linear regression analysis. Stepwise regression was used for the analysis, with a test level of (α = 0.05) for the introduced variables and (α = 0.1) for the excluded variables. Based on the results of the linear regression of compensation, the variables that had a significant effect on the determination of compensation, based on the magnitude of the absolute value of the standardized coefficients, were years, treatment attributes, and regions. (See Table 3)

**Table 3:** Linear regression analysis of compensation cases in medical damage liability disputes

	β	Beta	t	*p* value
Constant	5.679		36.184	< 0.001***
2015	-0.369	-0.256	-5.636	< 0.001***
2016	-0.275	-0.168	-4.109	< 0.001***
2017	-0.229	-0.116	-3.088	0.002**
2018	-0.224	-0.103	-2.669	0.008**
2019	-0.364	-0.172	-4.386	< 0.001**
2020	-0.221	-0.113	-2.756	0.006**
Eastern	-0.137	-0.115	-2.668	0.008**
Middle	-0.074	-0.061	-1.422	0.155
Township health center	-0.133	-0.052	-1.693	0.091
County Hospital	-0.027	-0.018	-0.587	-0.557
Low cost-effectiveness of treatment	-0.578	-0.138	-4.314	< 0.001***
Poor treatment effect	-0.594	-0.250	-7.832	< 0.001***
Complications of treatment	-0.488	-0.230	-6.998	< 0.001***
Treatment leading to disability	-0.169	-0.140	-4.234	< 0.001***
First trial	-0.206	-0.148	-1.381	0.168
Second trial	-0.150	-0.111	-1.080	0.280

Note: ***、** and *indicate the significant *p* value at the 0.1%、1% and 5%

## Discussion

### Years and regions distribution of COMDLD

The results of the study show that medical damage liability disputes are taken seriously by society and that COMDLD shows a general upward trend with the year, which is consistent with the findings of Zhou L and Zeng Y et al. [26, 27]. One of the possible reasons for this is that the technical level of medical institutions needs to be improved and the awareness of service management needs to be optimized. Second, the deep-rooted expectation gap between doctors and patients may lead to misunderstandings when patients encounter unexpected medical outcomes, which may lead to medical disputes [28]. For example, in the Tao Yong case, a well-known Chinese ophthalmologist brought hope of sight to patient Cui, only to have the patient slashed and injured because he was dissatisfied with the outcome of the surgery. The third reason may be due to the introduction and improvement of relevant laws and regulations, such as the Regulations on the Treatment of Medical Accidents and its supporting documents, the Interpretation of the Supreme People’s Court on Several Issues Concerning the Application of Law in Hearing Cases of Compensation for Personal Damage, and the Regulations on the Prevention and Treatment of Medical Disputes, coupled with the publicity on media platforms, which have raised patients’ awareness of the safety of using legal weapons to defend their rights. Other possible causes are not excluded.

In terms of regional distribution, COMDLD is distributed in all regions of China, and shows an uneven distribution, with more distribution in the Eastern and Middle regions. The reasons for this may be as follows; firstly, the Eastern and Middle regions are more economically developed, the population is highly educated, legal knowledge is well publicized, and awareness of rights is higher. Therefore, when suffering from medical damages, they usually resort to legal means to defend their legal rights promptly. Secondly, the distribution of medical resources in China is extremely unbalanced, with medical resources nationwide being distributed mainly in the economically developed eastern provinces and the densely populated Middle provinces [29, 30]. As a result, patients with difficult medical conditions from all over the country will come to medical institutions in the eastern and Middle regions from all over the world in search of better medical resources, with diversified diseases and complications, making it more difficult to cure and the higher the risk of medical accidents. In addition, it may be related to the degree of population concentration. The Eastern and Middle are densely populated, while the Western region is sparsely populated. The larger the population, the greater the number and frequency of visits, and the greater the likelihood of doctor-patient disputes.

### Distribution of types of hospitals, patients’ purposes for appeal, and trial procedures in cases of CMDLD

The results of this study showed that the highest incidence of medical damage liability cases in China was found in tertiary hospitals (city/provincial hospitals), which is consistent with the findings of previous studies [27]. Possible reasons for this are as follows: firstly, the Chinese healthcare system is still flawed. At present, China’s hierarchical medical system is still in its infancy and is not yet sound [31]. The higher-ranked hospitals have a high load and the lower-ranked hospitals have not yet played a better role in diverting traffic. With the rise in economic standards, people generally want better medical resources and flock to tertiary hospitals with better medical technology for both major and minor illnesses. These are mainly city/provincial hospitals with a high volume of patient visits and more medical services provided by the hospitals and are therefore also more prone to medical dispute incidents. Secondly, the higher the level of hospital attendance in China, the higher the workload of medical staff and the lack of effective communication with patients. The short duration and low content of doctor-patient communication prevent patients from obtaining sufficient information, and language and cultural differences also act as barriers to information exchange between doctors and patients [32]. In the event of adverse outcomes or a prognosis that does not meet the patient’s expectations, the patient may consider the medical practitioner to be irresponsible and negligent, making him or her vulnerable to medical disputes. Thirdly, patients generally spend more at higher-level hospitals than at lower-level hospitals and have higher expectations of the outcome of their treatment. For example, the reimbursement rate for residents’ health insurance is 80% for first-class hospitals, 60% for second-class hospitals, and 50% for third-class hospitals. However, as most of the difficult cases are concentrated in higher level medical institutions such as city/provincial level, the treatment is difficult and the risk of treatment is high, therefore the proportion of medical incidents during the treatment is also higher compared to other medical institutions.

The results of this study show that there is a high rate of appeals in the second trial and a high rate of hospital compensation. The data of this study shows that the largest number of cases of medical damage liability dispute compensation in the second trial, 701 cases, but the largest number of cases in the first instance with a median financial compensation of 183,497.90 yuan, and a hospital compensation rate of 52.46%. The reasons for this are as follows: firstly, China’s Tort Liability Law provides that where a patient suffers damage in the course of medical treatment activities and the medical institution and its medical staff are at fault, the medical institution shall bear the liability for medical damage; secondly, the patient’s litigation expectations were too high and there was a huge contrast between its and the realized value. Patients, not being satisfied with the amount of the judgment, will in turn bring it up again to defend their rights and interests.

### Different influencing factors of CMDLD

This study conducted a linear regression analysis on the factors influencing medical damage liability compensation, and the results showed that the outcome of medical damage liability disputes was associated with all three factors: years, regions, and treatment attributes.

The results of this study showed that the median amount of compensation for medical damage liability disputes had significant differences between years, with medical damage liability damages fluctuating but generally showing an upward trend. This is consistent with the findings of Zhou L et al. [27]. 2021 had the highest median amount of compensation for medical damage liability disputes at 191,897.83 yuan. Possible reasons for this are as follows: firstly, people’s increasing awareness of the legal system and their awareness of defending their legitimate rights and interests; in addition, the improvement of the overall legal system in society and the increased protection of vulnerable groups is another important reason for this status quo. Finally, in the past, China’s civil tort compensation was based on the principle of “filling in the losses”, with the tortfeasor paying as much as the right holder lost. Statistics from relevant scholars show that there can be a difference of 2.56 times in death compensation between urban and rural residents. Since the Supreme People’s Court issued the “Notice on the Authorisation of the Pilot Project on the Unification of Urban and Rural Standards for Personal Damage Compensation” in September 2019, the amount of personal damage compensation for rural residents has been greatly increased, which has also contributed to the increase in the overall level of compensation for medical damage liability disputes.

The results of this study show that the median amount of compensation for medical damage liability disputes varies greatly between regions, with the Eastern and Middle regions accounting for a larger proportion of medical damage liability disputes and a higher median amount of compensation than the Western regions. The reasons for this may be as follows: one reason is that the eastern and Middle regions have a relatively higher level of medical technology and receive more critically ill patients, which can result in more serious consequences of medical damage if not treated properly; a possible reason is that the costs of medical treatment, accommodation, transportation and per capita wage levels are higher in the eastern and Middle regions than in the Western regions, and these costs are all linked to the cost of medical damage compensation; the third possible reason is that there is a big difference in the level of medical care between the Eastern and Middle regions and the Western regions of China, and the standard of medical duty of care for medical personnel in the Eastern and Middle regions is higher than that in the Western regions, so that the amount of financial compensation incurred in the event of a medical injury is also higher.

The results of this study show that the more serious the consequences of the damage caused by the treatment, the higher the CMDLD. In the data of this study, medical treatment resulted in the highest number of cases of death, 399, and the highest compensation, with a median compensation of 186,300.00 yuan. The levels of medical malpractice are explained in China’s laws and regulations on medical malpractice. Firstly, Article 4 of the Regulations on the Treatment of Medical Accidents, which came into force in China on 1 September 2002, provides that the classification of medical accidents is based on “the degree of damage caused to the patient’s person”. This regulation classifies medical accidents into four levels and provides a reference standard for the technical identification of medical accidents. Secondly, the Health Law specifies that compensation for medical incidents should take into account the level of medical incidents, and the more serious the medical incident, the higher the amount of compensation. Third, Article 12 of the Interpretation of the Supreme People’s Court on Several Issues Concerning the Application of Law in Hearing Cases of Compensation for Personal Damage stipulates that the disability compensation shall be calculated by the degree of the victim’s loss of working capacity or the level of disability, by the standard of the per capita disposable income of urban residents in the previous year in the locality of the court under appeal, and shall be calculated based on twenty years from the date of determination of disability. Therefore, the more serious the consequences of the treatment damage, the higher the amount of compensation.

### Limitations of this study

The limitation of this study is that the data comes from public databases, and in the process of data collection, it is more difficult to take effective remedial measures for the undisclosed and missing key information in the cases of medical damage liability disputes, which may have a certain impact on the results of the study. Secondly, this paper focuses on analyzing the influencing factors of compensation for medical damage liability disputes, and fails to reasonably extract and summarize the reasons for the occurrence of medical damage liability disputes in the cases, so the research needs to be in-depth. In addition, this study puts forward relevant suggestions for the main factors of compensation for medical damage liability disputes, which provides a reference for the risk management of doctor-patient relationship. However, the inclusion of only data on medical damage liability dispute cases in China may limit the generalization of the research results.

## Conclusions

In general, with the frequent occurrence of medical damage liability disputes and the increasing amount of CMDLD, the developed Eastern and Middle regions and municipal/provincial medical institutions have become the high-incidence areas for medical damage compensation, and the compensation and appeal rates of cases remain high, leading to the deteriorating DPR and the squeezing and wasting of judicial resources, which seriously threatens the development of China’s health and the harmony and stability of society. To ease the DPR, improve the quality of medical care and build a harmonious medical environment, we make the following recommendations.

First, the unreasonable allocation of medical resources is an important factor leading to the low quality of medical services and obvious regional differences in compensation for medical damage liability disputes. The government should promote the optimization and adjustment of medical resources to achieve resource sharing on a large regional scale, improve allocation efficiency, and narrow the gap between urban and rural areas and regions. Secondly, it can promote the construction of medical consortia through the expansion and sinking of high-quality resources, and continuously improve the comprehensive capacity of county hospitals. This will enhance the accessibility of medical services in primary medical institutions, reduce the operational pressure of large hospitals, and ensure the improvement of medical services at all levels of hospitals, thus achieving the result of reducing the occurrence of medical damage incidents in high-level hospitals. Finally, the cost of medical litigation is reduced through the development of a third-party negotiation mechanism for medical disputes. At present, third-party mediation of medical disputes has received widespread attention [33, 34]. Its high efficiency, acceptability, and low cost make up for the drawbacks of traditional settlement methods. However, there is still a need to improve the legal system related to the third-party negotiation mechanism, establish a professional talent team, and build a multi-party linkage and cooperation mechanism, so that it can improve the quality and efficiency of resolving medical damage liability disputes.

## Data Availability

The dataset analyzed in this study is only available when successfully registered using one’s cell phone number, and access to the dataset should be requested from the China Judgment Online (https://wenshu.court.gov.cn/). The data used and/or analyzed during the current study is available from the corresponding author on reasonable request.

## Abbreviations

CMDLD: Compensation for medical damage liability disputes
DPR: Doctor-patient relationships
COMDLD: Cases of medical damage liability disputes
